# Intra-Individual Variability of Surface Electromyography in Front Crawl Swimming

**DOI:** 10.1371/journal.pone.0144998

**Published:** 2015-12-16

**Authors:** Jonas Martens, Daniel Daly, Kevin Deschamps, Ricardo Jorge Pinto Fernandes, Filip Staes

**Affiliations:** 1 Department of Kinesiology, KU Leuven, Leuven, Belgium; 2 Department of Rehabilitation Sciences, KU Leuven, Leuven, Belgium; 3 Centre of Research, Education, Innovation and Intervention in Sport, Faculty of Sport, University of Porto and Porto Biomechanics Laboratory, University of Porto, Porto, Portugal; Universite de Nantes, FRANCE

## Abstract

The variability of electromyographic (EMG) recordings between and within participants is a complex problem, rarely studied in swimming. The importance of signal normalization has long been recognized, but the method used might influence variability. The aims of this study were to: (i) assess the intra-individual variability of the EMG signal in highly skilled front crawl swimmers, (ii) determine the influence of two methods of both amplitude and time normalization of the EMG signal on intra-individual variability and of time normalization on muscle activity level and (iii) describe the muscle activity, normalized using MVIC, in relation to upper limb crawl stroke movements. Muscle activity of rectus abdominis and deltoideus medialis was recorded using wireless surface EMG in 15 adult male competitive swimmers during three trials of 12.5 m front crawl at maximal speed without breathing. Two full upper limb cycles were analyzed from each of the swimming trials, resulting in six full cycles used for the intra-individual variability assessment, quantified with the coefficient of variation (CV), coefficient of quartile variation (CQV) and the variance ratio (VR). The results of this study support previous findings on EMG patterns of deltoideus medialis and rectus abdominis as prime mover during the recovery (45% activity relative to MVIC), and stabilizer of the trunk during the pull (14.5% activity) respectively. The intra-individual variability was lower (VR of 0.34–0.47) when compared to other cyclic movements. No meaningful differences were found between variability measures CV or VR when applying either of the amplitude or the time normalization methods. In addition to reporting the mean amplitude and standard deviation, future EMG studies in swimming should also report the intra-individual variability, preferably using VR as it is independent of peak amplitude, provides a good measure of repeatability and is insensitive to mean EMG amplitude and the degree of smoothing applied.

## Introduction

The intra and inter individual variability of surface electromyographic (sEMG) recordings is a complex problem previously examined in cyclic movements such as gait [1–4], running [5] and cycling [6,7]. Several major questions have been addressed on this topic including consistency in recordings of the same movement repeated several times by one individual [8] and the variability of these recordings between individuals performing the same movement. In the 50 year history of electromyographic (EMG) research in swimming, one study has evaluated the intra-individual variability of EMG recordings [9]. In this study, “ten swimmers of different levels of ability” were tested over several trials but the results of one muscle (triceps brachii) were presented for only one swimmer [10]. It was concluded that “the repeatability of swimming movements by highly skilled swimmers appears to be exceptionally high, as measured by both duration and quantified electromyography” and that “a limited number of stroke cycles may therefore be accepted as valuable information.” Caution is warranted as conclusions were based on limited results with no detailed information on the testing protocol. Yet all conclusions in EMG studies in swimming investigating amplitude have been based on single stroke cycles from one or more participants.

The importance of amplitude normalization of the EMG signal has long been recognized as essential when comparing EMG patterns across participants, trials and muscles [8,11]. Nevertheless, the fact that the reduction of both the intra and inter-individual variability is dependent on the normalization method used cannot be ignored [11,12]. The normalization method used is subject to debate both in swimming EMG research and beyond [13]. EMG studies in swimming have used both the maximal voluntary isometric contraction (MVIC) method (47% of studies) and the dynamic peak method (23%). The remaining 30% of studies used no normalization [9]. For normalization purposes, the EMG from a specific task or event is divided by the EMG from a reference contraction of the same muscle resulting in a relative measure (%) [13]. In the MVIC method, introduced in swimming by Barthels et al. [14], each data point is divided by the peak EMG from a maximum voluntary isometric contraction performed on dry land. In the dynamic peak method, introduced in swimming by Rouard and Billat [15], each data point is divided by the peak value recorded during the (swimming) trial itself [12]. This is different from the MVIC method where one value is used to normalize all cycles. As dynamic peaks can differ between cycles, this can influence the variability measures. Two things can happen. If the shape of the curves is similar in all cycles, a normalization of each cycle to its own peak will reduce the inter-curve variability since amplitude differences will be leveled out. This will therefore reduce variability. If the shape of the curves is dissimilar, this approach can increase inter-curve variability. At present, the effect of both approaches on variability measures has never been investigated in swimming.

A second form of normalization to be considered is time normalization as precise iteration of cycle duration is extremely unlikely in human movement. While it is common practice in gait analysis to consider the start and stop of each cycle as the 0 and 100% on the normalized time scale [16], it has never been done in EMG research in swimming. In addition, to the author’s knowledge, no studies have reported on multi event time normalization, where the cycle is normalized not only for start and end of each cycle, but for intermediate key moments within the cycle as well.

In the assessment of the intra-individual variability of EMG amplitude and time patterns, various variability measures have been used (calculation formulas of these measures are presented in Methods). In running, for instance, mean, standard deviation (SD) and the mean coefficient of variation (CV), as well as CV expressed over the running cycle, were proposed [5]. However, CV is influenced by the mean EMG value (the denominator in the CV formula) and might overestimate variability in the sectors in which muscle activity is non-existing or weak [17]. To counter this shortcoming, the use of the variance ratio (VR) has been proposed by Hershler and Milner [18] and applied in gait analysis [1–4]. Granata et al. [4] also calculated CV for comparison with previous literature. Knutson et al. [11] stated that “in the case of EMG, when waveforms are similar, the VR tends toward zero and when the waveforms are dissimilar, the VR tends toward one”, with this measure being independent of peak amplitude, providing a good measure of repeatability in the overall wave shapes [3]. Furthermore, it is insensitive to mean sEMG amplitude and the degree of smoothing applied to the data [19], and therefore is generally accepted as an excellent way to document the intra-individual variability [20].

As stated above, research has shown that the normalization method used affects variability. The intra-individual variability of the activation level of the gastrocnemius muscle in a cyclic single leg task on a balance board, as assessed with the VR, was found to be highest when the MVIC was used [11]. A lower intra-individual CV was found using the dynamic peak method [11]. Conversely, the intra-individual variability of gluteus medius activity in hip abduction exercises, expressed by CV, was found to be lower when using the MVIC compared to the dynamic peak method [12]. In a review on normalization methods, Burden [13] concluded that the MVIC method is preferable because of lower intra-individual variability. Ball and Scurr [21] stated however that this statement might be true for low-velocity muscle actions but that activities that require a high-velocity exertion such as fast running or jumping might need an alternative approach (e.g. dynamic peak method). Muscle actions in swimming are slower than those in high-velocity movements, but there has been no investigation of the influence of normalization methods for EMG recordings in swimming.

In contrast to the above referred studies, the sole variability measures reported in swimming EMG literature were the mean (normalized) activation and SD. In a study on the average activation pattern of shoulder muscles during front crawl in “20 collegiate and master level competitive swimmers”, SDs expressed as a percentage of the MVIC ranged between 33% and 52% [22]. In studying a much more homogenous group of nine swimmers, different (latissimus dorsi) to even opposite (anterior deltoid) patterns of mean amplitude were found with high inter-individual variability reported as SD [23]. When examining studies on EMG in swimming, variability around the mean is always large, leading to the assumption that not one general muscle activation pattern exists for all swimmers despite almost all researchers assuming this to be a fact. Before addressing the issue of the inter-individual variability however, the intra-individual variability should be analyzed. Apart from the study previously mentioned [10], this has never been done in swimming EMG related research and, considering the level of intra-individual variability generally observed in other cyclic movements, this is deemed necessary. Therefore, the aims of this study were to: (i) assess the intra-individual variability of the EMG signal of bilaterally measured rectus abdominis and deltoideus medialis in highly skilled front crawl swimmers, (ii) determine the influence of two methods of both amplitude and time normalization of the EMG signal on several intra-individual variability measures and of time normalization on muscle activity level and (iii) describe the muscle activity, normalized using MVIC, in relation to upper limb crawl stroke movements.

## Methods

### Participants

Data were obtained from 15 adult male competitive swimmers whose descriptive characteristics are shown in Table 1. None of the participants was suffering from any type of injury. All participants were informed about the goal and the methods of the study and gave their written consent to conditions approved by the ethical committee of KU Leuven (S55199) and in accordance with the Declaration of Helsinki.

**Table 1:** Participant descriptive statistics (n = 15).

	Mean	SD
Age (yrs)	21.26	2.24
Height (cm)	186.55	5.50
Mass (kg)	79.10	7.98
Adipose tissue (%)	13.49	4.71
Arm span (cm)	193.77	6.87
Best time 100 m front crawl (s)	54.72	1.93
Level (FINA points)	634.13	68.98
Number of years of competitive swimming experience (yrs)	11.93	3.24

### Participant preparation

To keep skin impedance low, the site for electrode placement was prepared by shaving, gently abrading the skin using sandpaper and cleaning with 70% isopropyl alcohol. Surface electrodes were positioned on the left and right deltoideus medialis and left and right rectus abdominis following the European Recommendations for Surface Electromyography (SENIAM) [24] and Cram and Kasman [8] respectively. These muscles were chosen based on their function as a prime mover (deltoideus medialis) and a stabilizer (rectus abdominis) in crawl swimming [9]. Waterproof taping of the EMG units was achieved by carefully covering the unit with Opsite Flexifix clear film (Smith & Nephew®, London, UK) and then covering the edges with sports tape.

### Experimental protocol

After positioning and waterproofing the EMG electrodes, participants were placed in the optimal muscle testing position [25], and performed two maximum voluntary isometric contractions of 5 s (with 2 min rest interval). Strong verbal encouragement was given to the participants and the single maximum value over the two measurements was defined for normalization. Subsequently, each participant swam 25 m in front crawl with a push-off water start with the first 12.5 m accelerating to their maximal speed (breathing was allowed) and maintaining maximal speed during the last 12.5 m (without breathing). The swimmers repeated this protocol as many times as necessary to achieve three successful trials collecting EMG recordings of all muscles. In between trials, there was a rest interval of 10 min to eliminate fatigue.

### Data collection

EMG was recorded using four channels of a five channel wireless electromyograph (KINE®, KINE Ltd., Hafnarfjördur, Iceland) with an input impedance of 10 GΩ, a common mode rejection ratio of 110 dB, a signal-to-noise ratio of 60 dB, a differential detection mode and a built in A/D converter of 10 bit with a range of 4 mV, resulting in a sensitivity of 4 μV. The Al/AgCl electrodes (including the reference electrode) were built into a single unit of 26 g and measuring dimensions of 56 mm length, 46 mm width and 16 mm height in a fixed triode configuration with a center-to-center inter-electrode distance of 20 mm EMG signals were streamed live to a laptop, but when the electrodes were submerged, live signal was lost. In these cases, the system automatically stored the data in a built in internal memory of maximal 7 min. The data was recollected when live connection was restored when the swimmer came out of the water after each trial. EMG data was collected live or recollected from the internal memory at a sample rate of 1600 samples per second with KINE Pro software [26]. Two underwater stationary 50 Hz video cameras (Sony Handycam HDR-HC9) were used to record the swimmers’ movements in their frontal and sagittal planes in the final 12.5 m window of each swim trial. Two upper limb cycles were captured allowing the upper limb movements to be linked to the EMG data. Cameras were synchronized using Dartfish Software Prosuite 6.0 and to the EMG signal via a LED light connected to the EMG equipment illuminating at the start of the EMG recording.

### Data processing

#### Kinematic data processing

**Fig 1:**
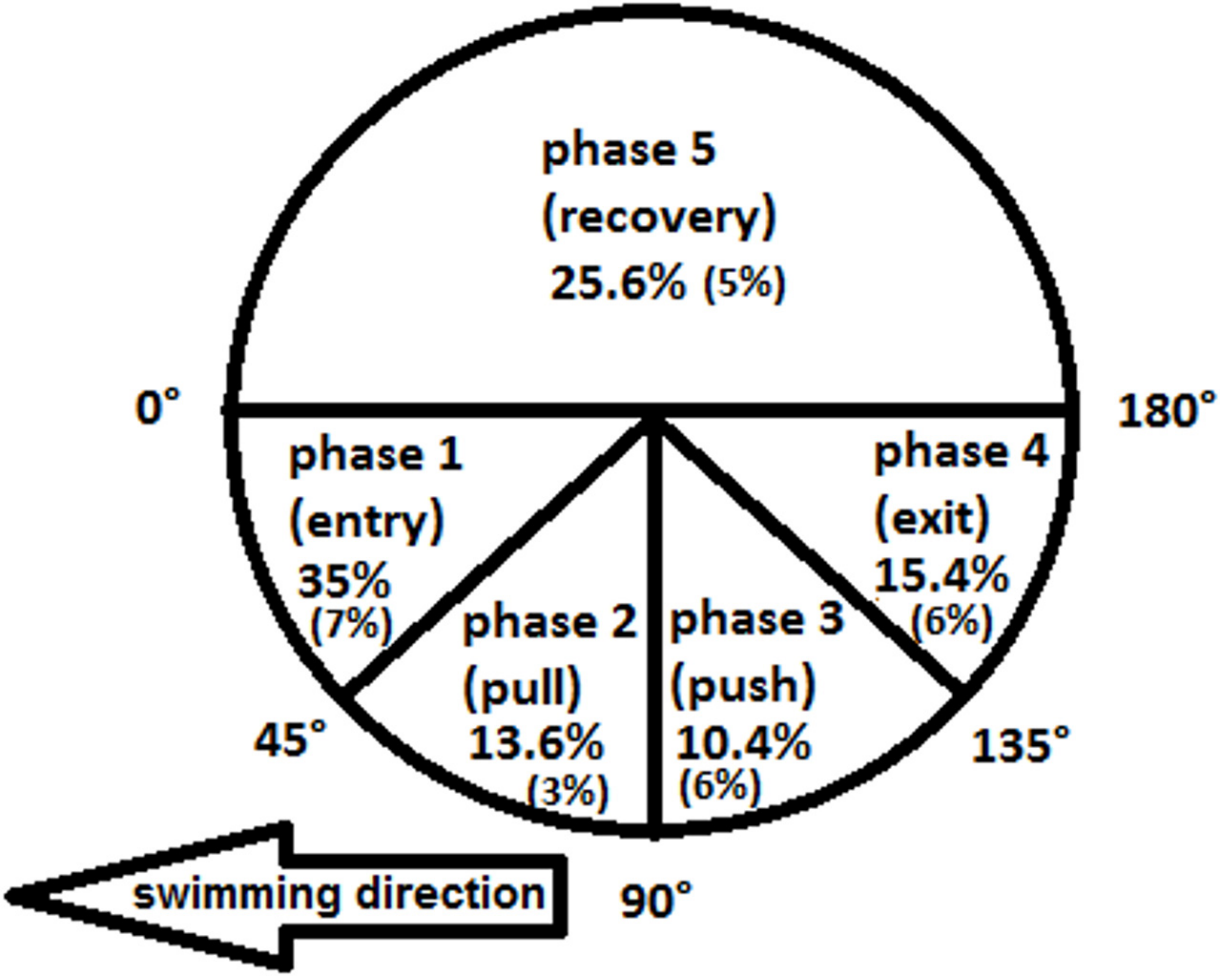
Mean decomposition (and SD) of the percentages of the total time of the upper limb cycle in the sagittal plane for the five different phases, defined by the upper limb angles to the horizontal (adapted from Rouard & Billat, 1990).

EMG was analyzed for two upper limb cycles for both left and right upper limb in the final 12 m (when swimming velocity was maximal) from each of the three swimming trials, resulting in the recording of six full cycles for both upper limbs. Arm cycle phases were defined based on angle of upper limb (the line from shoulder to wrist) in relation to the horizontal in the sagittal plane [15,27]: from 0 to 45° (entry), from 45 to 90° (pull), from 90 to 135° (push), from 135 to 180° (exit) and from 180 to 360° (recovery) using Dartfish Software Prosuite 6.0 (Fig 1). The clean swimming velocity in each trial was obtained by digitizing the upper rim of the swimsuit in the last 12 m of each trial and then averaging.

#### EMG data processing

KINE Pro software was used for filtering with a 2^nd^ order Butterworth filter with cut-off frequencies of 20 Hz– 800 Hz and slopes of -12 dB/octave, full-wave rectification and integration with an Average Rectified Value (ARV) with a moving window of 0.025 s and a step rate of one sample of the raw EMG signals.

#### Amplitude normalization

Two amplitude normalization techniques were applied using ACEP Manager: (i) the MVIC normalization method (the highest value, in micro volts, found during the MVIC was considered 100%) and (ii) the dynamic peak normalization method as proposed by Bolgla & Uhl [12] (the highest value found during each swimming cycle was considered 100% for that specific cycle).

#### Time normalization

As upper limb cycles had different phase timing both within, as well as between swimmers, time normalization is necessary and multi-event synchronization was performed for all selected cycles (left and right separately) using an in-house build Matlab 2012a ® based software framework (ACEP*Manager*). For each measurement, the complete cycle was determined by a start and stop event (hand entry at 0 and 360°, respectively) and then mapped to a 1000 point (promille) time scale, similar to the time normalization method used for gait analysis [5], resulting in a first time normalization, referred to as the “non-event synchronized” condition. In addition, intermediate events (the angles determining the cycle phases) were synchronized to the mean timing of that event of all swimmers using the interp1 Matlab 2012 function with the default linear interpolation method. After this operation, the durations of the complete cycle, as well as each phase, were equal for all cycles, resulting in a second time normalization, referred to as the “event-synchronized” condition.

### Data analysis

To study the intra-individual variability, the mean EMG signals and SDs for the six cycles of each participant for all four muscles were calculated for the entire cycle and for each phase. One dimensional CV (Eq 1) was calculated as follows, permitting the comparison of the variability of a data set with a larger and a smaller mean and SD [2]:

$$CV\;=\;\frac{\sqrt{\frac{1}{k}\;\sum_{i=1}^{k}\sigma_{i}{}^{2}}}{\frac{1}{k}\;\sum_{i=1}^{k}\left|\bar{X_{i}}\right|}$$ (1)

where *k* was the number of intervals over the cycle (i.e. 1000), ${\bar{X}}_{i}$ was the mean of the EMG values at the *i*th interval calculated over the six cycles and *σ*_*i*_ was the SD of the EMG values about ${\bar{X}}_{i}$ calculated over the six cycles.

When taking into account a time perspective in an attempt to present variability in two dimensions, CV (Eq 2) at the *i*th time interval was calculated as:

$${CV}_{i}\;=\;\frac{\sigma_{i}}{\bar{X_{i}}}$$ (2)

The mean value of 1000 *CV*_*i*_’s was defined as “mean CV”.

VR (Eq 3) was calculated as an extra one dimensional measure of variability [18] as follows:

$$VR\;=\;\frac{\sum_{i=1}^{k}\sum_{j=1}^{n}(X_{ij}-{\bar{X}}_{i})\;{}^{2}/k(n-1)}{\sum_{i=1}^{k}\sum_{j=1}^{n}(X_{ij}-\bar{X})\;{}^{2}/(kn-1)}$$ (3)

where *k* was the number of intervals over the cycle (i.e. 1000), *n* was the number of cycles (i.e. 6), *X*_*ij*_ was the EMG value at the *i*th interval for the *j*th cycle, ${\bar{X}}_{i}$ was the mean of the EMG values at the *i*th time interval over *j* cycles and $\bar{X}$ was the mean of the EMG values, i.e. $\bar{X}\;=\;\frac{1}{k}\;\sum_{i=1}^{k}\bar{X_{i}}$.

Finally, the coefficient of quartile variation (CQV in Eq 4) was calculated as an extra measure of variability in two dimensions, where Q1 was the 25^th^ percentile and Q3 the 75^th^ percentile of the six EMG values at a given time interval [28].

$$CQV\;=\;\frac{\left(Q3-Q1\right)}{\left(Q3+\;Q1\right)}$$ (4)

Statistical differences between one-dimensional variability measures in the two time (event and non-event synchronized) and two amplitude (MVIC and dynamic peak) normalization methods, the two muscles and left and right were assessed using a two-tailed paired T-test (P < 0.05) as previously used in studies with similar designs and aims [2,29] in Microsoft Excel 2010. Furthermore, a Bonferroni-Holm correction for multiple testing was implemented considering the four pairwise comparisons. Statistical differences between mean muscle activity level were only assessed within each amplitude normalization method since a comparison of EMG amplitude between the two amplitude normalization methods is not appropriate. The denominator in both methods has a physiologically different origin. In contrast with the MVIC method, the dynamic peak method does not provide information on the magnitude of muscle activation. As only the one dimensional generated data were statistically analyzed, a mean value over the 15 swimmers was calculated for each measure for inferential statistical analysis.

## Results

Fig 1 shows the average duration of each of the five upper limb phases (sagittal plane), defined by the upper limb angles to the horizontal, relative to the full cycle.

**Fig 2:**
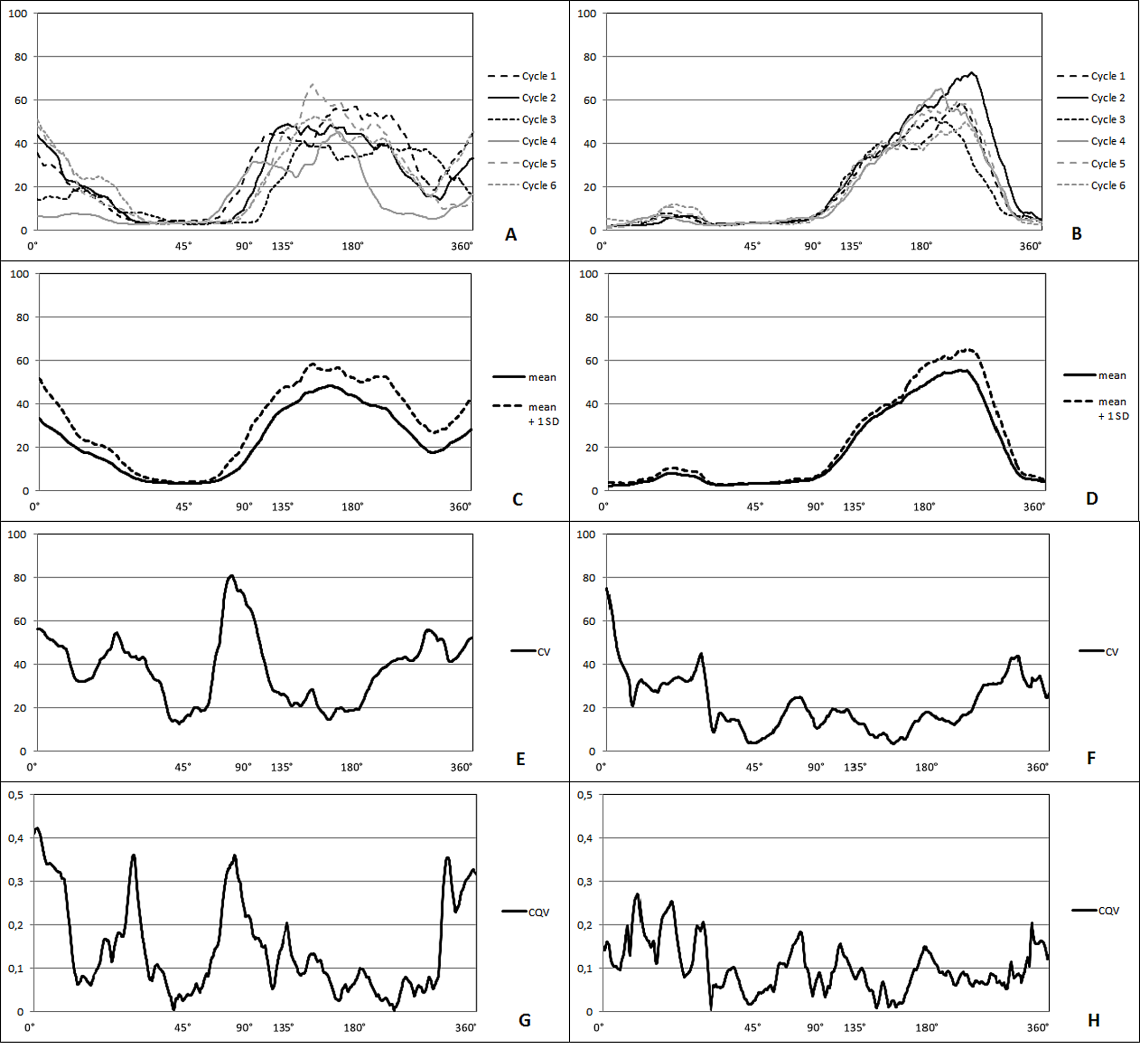
Muscle activity (%) of the right deltoideus medialis of six cycles of a swimmer with a high (A) and a low variance ratio (B). Mean activation and SD (C and D), coefficient of variation (E and F) and coefficient of quartile variation (G and H) for the swimmer with high (C, E, G) and low (D, F, H) variance ratio are shown.

The first aim of this study was to assess the intra-individual variability of the EMG signal of bilaterally measured rectus abdominis and deltoideus medialis in highly skilled front crawl swimmers. Examples of the muscle activity and variability of the right deltoideus medialis in six cycles of two swimmers normalized to MVIC are presented in Fig 2, comparing the swimmer with the highest intra-individual variability expressed in terms of VR (0.50) of all participants for this muscle to the swimmer with the lowest VR (0.19).

Table 2 presents the mean muscle activity levels (amplitude) and intra-individual variability of the normalized (both to amplitude and time) EMGs from the two muscles evaluated, averaged over all participants. Since no differences were found in any of the selected parameters between the left and right side of each muscle, data were pooled for both deltoideus medialis and rectus abdominis. Rectus abdominis showed more intra-individual variability for all variability measures compared to deltoideus medialis.

**Table 2:** Activity levels (%) and intra-individual variability of swimming EMG normalized using the MVIC and the dynamic peak (DP) method averaged over 15 swimmers for both event (ES) and non-event synchronized (NES). DM = deltoideus medialis, RA = rectus abdominis, CV = coefficient of variation, VR = variance ratio.

			MVIC		DP	
			ES	NES	ES	NES
Activity level	DM	Mean (SD)	28.14 (6.86)	27.95 (6.59)	25.27 (5.54)	25.12 (5.38)
(%)	RA	Mean (SD)	8.03 (2.63)	7.98 (2.56)	16.35 (4.21)	16.34 (4.33)
Mean CV (%)	DM	Mean (SD)	46.58 (6.86) ^a^	46.81 (5.73) ^b^	47.26 (5.77) ^a^	47.42 (5.72) ^b^
	RA	Mean (SD)	53.72 (10.79) ^a^	52.85 (9.97) ^b^	56.22 (11.22) ^a^	55.69 (10.79) ^b^
CV	DM	Mean (SD)	0.52 (0.07)	0.52 (0.07)	0.52 (0.07)	0.52 (0.07)
	RA	Mean (SD)	0.76 (0.17)	0.76 (0.17)	0.74 (0.16)	0.74 (0.17)
VR	DM	Mean (SD)	0.34 (0.09)	0.35 (0.09)	0.35 (0.09)	0.35 (0.09)
	RA	Mean (SD)	0.46 (0.15)	0.46 (0.15)	0.46 (0.15)	0.46 (0.15)

^a^ significant differences in mean CV between the MVIC and the dynamic peak method in the event synchronized condition

^b^ in the non-synchronized condition.

The second aim of this study was to determine the influence of amplitude normalization of the EMG signal on several variability measures. Differences were found in mean CV between the MVIC and dynamic peak method. When determining the influence of time normalization of the EMG signal on variability measures and muscle activity level, no differences were found between event synchronized and non-event synchronized data.

A final aim of this study was to describe the muscle activity in relation to upper limb movements. These results are presented in Table 3. Mean activity is highest for the deltoideus medialis during the exit and recovery phases and for the rectus abdominis during the pull and push phases. Furthermore, deltoideus medialis showed higher mean activity during its most active phase (45.6%) when normalized to MVIC as compared to rectus abdominis (14.5%).

**Table 3:** Mean activity level of swimming EMGs normalized (%) using the MVIC, event synchronized method averaged over 15 swimmers per upper limb phase. DM = deltoideus medialis, RA = rectus abdominis.

		Left DM	Right DM	Left RA	Right RA
Entry	Mean (SD)	15.45 (8.65)	17.36 (11.02)	6.82 (2.55)	7.83 (4.11)
Pull	Mean (SD)	7.00 (5.49)	8.45 (5.94)	13.89 (7.69)	14.55 (10.87)
Push	Mean (SD)	34.73 (10.86)	36.00 (11.25)	9.53 (4.54)	10.93 (5.85)
Exit	Mean (SD)	43.95 (11.20)	45.64 (8.43)	3.50 (2.16)	3.94 (2.69)
Recovery	Mean (SD)	44.81 (12.92)	39.28 (11.27)	7.87 (4.51)	6.77 (4.90)

## Discussion

The first aim of this study was to assess the intra-individual variability of the EMG signal in highly skilled swimmers. All one dimensional variability measures differed between the two muscles (Table 2) with a larger intra-individual variability in rectus abdominis. No significant difference was found between left and right sides of either muscle. The latter does not necessarily mean that no differences existed in EMG patterns, as can be seen in Fig 2, but indicates that these swimmers were equally stable in their EMG pattern on both sides. Furthermore, Fig 2 (panels E and F) shows the high intra-individual variability when using CV in the sectors in which the muscle showed low activity [17]. CQV (panels G and H) appears to be a better variability measure with a time perspective (when presenting data in two dimensions) since SD and mean are not necessarily the most meaningful estimators of spread and location in skewed distributions such as EMG. Therefore, when sampling from nonnormal distributions, the coefficient of quartile variation may be preferred [28].

A potential source of muscle variability could have been the swimming velocity of the participants and its variability within the test situation. The 15 swimmers swam on average 1.85 m/s (SD 0.08 m/s; range 1.73–2.03 m/s) corresponding with a maximal effort when compared to their 100 m front crawl average best time (Table 1). The swimmers were able to maintain this velocity both within each trial (average SD of 10 velocity measures per swimmer within that 12.5 m: 0.12 m/s), as well as across the three trials (average difference between fastest and slowest trial 0.08 m/s; SD 0.1 m/s).

Since there is no literature available to compare the present results, our variability measures were compared to other cyclic activities and often other muscles. In studying the intra-individual variability in running using mean CV [5], average results ranged from 53–59% for the muscles tested and it was concluded that the intra-individual data were extremely stable. Mean CV values in the present study were even lower, leading to the same conclusion. When considering VR as a measure of variability, the values found in this swimming study were again lower than those found in gait where VR ranged from 0.51–0.76 [2]. One possible explanation might be the different type of muscle contraction, with more eccentric contraction seen during gait. However, no swimming study has ever investigated the type of muscle contraction and further study is needed to explain this phenomenon.

Table 3 showed lower activity values in rectus abdominis as compared to deltoideus medialis using the same normalization method (MVIC). In front crawl swimming, rectus abdominis acts more as a trunk stabilizer, requiring a submaximal muscular effort. It has been suggested that sufficient core stability is needed to balance forces generated by the upper and lower extremities in general [30,31], and specifically in swimming [32,33]. In front crawl swimming, the force generated by the upper limbs is largest when the lever is longest, namely in the pull and push upper limb phases. In the present study, the highest mean activity of rectus abdominis is found in these two phases (Table 3). This is consistent with the findings of Piette & Clarys [34]. The deltoideus medialis acts, in contrast to the rectus abdominis, as a prime mover of the upper limb [32]. The upper limb has a larger range of motion allowing the deltoideus medialis to reach more of its full potential. Swimmers reached a higher mean activity when normalized to the MVIC compared to rectus abdominis (Table 3). Corresponding to previous studies on the deltoideus medialis [22,35], the participants of this study showed highest activity in the final underwater propulsive phase (exit) and the recovery when the upper limb is abducted.

This study further aimed to determine the influence of two methods of amplitude normalization on several variability measures. Previous studies on land suggested that normalizing with the MVIC method is preferable because of lower intra-individual variability [13]. Ball and Scurr [21] stated that this might be true for low-velocity muscle actions but that activities requiring a high-velocity exertion might need an alternative approach, such as the dynamic peak method. This swimming study did not reveal meaningful differences between the MVIC and dynamic peak method except for the mean CV. This is possibly due to the fact that mean CV is most influenced by differences in mean activity [17]. Furthermore, the movement velocity in swimming lies somewhere between those high velocity sports activities and low velocity activities on dry land such as gait.

It should be noted that the amplitude normalization methods used in this study differed as the denominator for normalization changed between cycles for the dynamic peak method. This was not the case for the MVIC method. As stated in the introduction, this can influence the intra-individual variability. Though, in our sample and considering the most reliable one dimensional variability measure, VR, variability did not differ on average when using either method. Thus, although in theory the variance in the dynamic peaks could have influenced the variability, in our sample it did not. However, this does not imply that on an individual participant level there was no impact.

As this study found no influence of amplitude normalization method on variability, the choice for a certain amplitude normalization method in future swimming research should not be based on the issue of variability, but rather on other considerations. The existing literature on normalization points out that each method has its pros and cons and a choice for either one (or in some cases both) should be based on these and the purpose of the study and considering the downsides of the chosen normalization method. An inherent limitation associated with MVIC normalization is the assumption that the participants provide a maximal effort. The use of a combination of positioning the participants in a consistent, standardized manner, strong verbal encouragement and task familiarization facilitates however a participant’s ability to perform a reliable MVIC [12]. A much read criticism furthermore is that this method results in outputs during the actual task in excess of 100% [36,37], in particular during rapid contractions or muscle lengthening [13].

When using the dynamic peak method on the other hand, questions arise on using this method to compare EMGs between different trials, muscles or individuals. The denominator is obtained during the task under investigation. This means that modifications in technique for example would be reflected in the denominator as well as the numerator and therefore this normalization technique might not pick up changes in magnitude or patterning of EMGs [13]. Furthermore, using this method might make it impossible to detect differences between left and right when bilaterally studying certain muscles for example in swimmers with unilateral impairments or injuries. In retrospect, in a study examining the influence of the size of hand paddles in front crawl swimming [38], the MVIC method would have been more appropriate than the dynamic peak method used, since differences between the different conditions might have existed, but were masked by the fact that the denominator was obtained during the swimming trials with the different hand paddles. On the other hand, in a study aiming to describe the patterns of two arm muscles [39], the use of the dynamic peak method would have been preferred over the usage of the MVIC method since no comparisons or modifications of the swimming technique were made. Furthermore, the dynamic peak method is useful for example to extract muscle synergies or analyze the shape of EMG patterns.

Finally, in determining the influence of time normalization, no differences were found in any variability measure or muscle activity level between event synchronized and non-event synchronized time normalized data (Table 2). Future research should therefore use event synchronization as a time normalizing method since this permits a more precise link between the EMG and the kinematic data and better prospects to interpret findings in relation to the upper limb stroke phase.

Some limitations of this study should be considered. 50 Hz cameras were used as this type of equipment is a standard evaluation tool in current stroke analysis and the vast majority of EMG studies in swimming used 50 Hz cameras. In future research however, high speed cameras might be needed to obtain more detailed kinematic data. Secondly, only two muscles were examined, a typical stabilizing muscle and a typical prime mover in front crawl swimming. A larger selection of muscles would have enabled more generalization of the current findings. Furthermore, the CQV calculation was based on only six cycles. More cycles could be needed to use Q1 and Q3 as meaningful variables in this calculation. Finally, the measurements used for this intra-individual variability assessment were all made in one day. It would be interesting to see what the variability is when retesting the same swimmers on a second day.

With regard to the statistical analysis, the authors decided not to use a two way ANOVA repeated measures analysis. This would have been appropriate when interaction effects would have been under consideration. As this was not the aim of our study, other statistical procedures were used. Furthermore, using a two way ANOVA repeated measures analysis would also introduce the more strict assumption of constant variance and covariance.

## Conclusions

The results of this study showed that the intra-individual variability of a group of highly skilled swimmers is lower when compared to other cyclic movements, indicating that a very limited number of stroke cycles per swimmer is sufficient for amplitude analysis of muscle activity, and no meaningful differences were found in variability measures CV and VR between the amplitude and time normalization methods studied. Furthermore, this study supports previous findings on EMG patterns of deltoideus medialis and rectus abdominis as prime mover during the recovery, and stabilizer of the trunk respectively during front crawl swimming.

## Supporting Information

S1 File Dataset MVIC method Intra-individual variability of EMG in front crawl swimming. (ZIP)

S2 File Dataset Dynamic peak method Intra-individual variability of EMG in front crawl swimming. (ZIP)
